# Destructive effects of murine arthritogenic antibodies to type II collagen on cartilage explants *in vitro*

**DOI:** 10.1186/ar1766

**Published:** 2005-06-06

**Authors:** Duncan E Crombie, Muhammed Turer, Beltzane Biurrun Zuasti, Bayden Wood, Don McNaughton, Kutty Selva Nandakumar, Rikard Holmdahl, Marie-Paule Van Damme, Merrill J Rowley

**Affiliations:** 1 Department of Biochemistry and Molecular Biology, Monash University, Clayton, Victoria, Australia; 2 Centre for Biospectroscopy and School of Chemistry, Monash University, Clayton, Victoria, Australia; 3 Medical Inflammation Research, Lund University, Lund, Sweden

## Abstract

Certain monoclonal antibodies (mAbs) to type II collagen (CII) induce arthritis *in vivo *after passive transfer and have adverse effects on chondrocyte cultures and inhibit self assembly of collagen fibrils *in vitro*. We have examined whether such mAbs have detrimental effects on pre-existing cartilage. Bovine cartilage explants were cultured over 21 days in the presence of two arthritogenic mAbs to CII (CIIC1 or M2139), a non-arthritogenic mAb to CII (CIIF4) or a control mAb (GAD6). Penetration of cartilage by mAb was determined by immunofluorescence on frozen sections and correlated with changes to the extracellular matrix and chondrocytes by morphometric analysis of sections stained with toluidine blue. The effects of mAbs on matrix components were examined by Fourier transform infrared microspectroscopy (FTIRM). A possible role of Fc-binding was investigated using F(ab)_2 _from CIIC1. All three mAbs to CII penetrated the cartilage explants and CIIC1 and M2139, but not CIIF4, had adverse effects that included proteoglycan loss correlating with mAb penetration, the later development in cultures of an abnormal superficial cellular layer, and an increased proportion of empty chondrons. FTIRM showed depletion and denaturation of CII at the explant surface in the presence of CIIC1 or M2139, which paralleled proteoglycan loss. The effects of F(ab)_2 _were greater than those of intact CIIC1. Our results indicate that mAbs to CII can adversely affect preformed cartilage, and that the specific epitope on CII recognised by the mAb determines both arthritogenicity *in vivo *and adverse effects *in vitro*. We conclude that antibodies to CII can have pathogenic effects that are independent of inflammatory mediators or Fc-binding.

## Introduction

An experimental model of the human autoimmune disease rheumatoid arthritis (RA) is provided by collagen-induced arthritis (CIA), which is induced in animals after immunisation with type II collagen (CII) [1,2], a major component of articular cartilage. The ensuing autoimmune response includes the formation of antibodies to CII that, on transfer to naïve mice, induce acute and destructive arthritis [3,4]. Antibodies to CII are present in the sera and synovial fluid of patients with RA [5-7] and epitopes include those targeted by arthritogenic antibodies from mice with CIA [8]. Debate continues, however, on whether autoantibodies to CII in RA are actual contributors to the pathogenesis, or merely reflect a reaction to cartilage degradation. Although antibody-induced CIA can be transferred by combinations of mAbs [4,9], and also by certain single mAbs [4,10], not all mAbs to CII are arthritogenic, and arthritogenicity appears to be epitope specific [8]. We postulate that there are certain species of anti-CII autoantibodies that do cause cartilage damage by binding specifically to critical structural regions on collagen fibrils that are sites of interaction between CII and matrix components or chondrocytes. Favouring this, arthritogenic mAbs to CII both inhibit collagen fibrillogenesis *in vitro *[11] and adversely affect the cartilage matrix and chondrocyte morphology in chondrocyte cultures [12,13]. On the other hand, cartilage is an avascular tissue in which there is minimal collagen synthesis in adults [14]; moreover, antibodies penetrate cartilage so poorly [15] that they may not be capable of disrupting a pre-existing cartilage matrix.

Accordingly, we examined the effects of different mouse mAbs to CII on cultured cartilage explants and found that these not only did penetrate and react with CII, but also had disruptive effects on a pre-established cartilage matrix. To help identify changes in the cartilage matrix we used Fourier transform infrared microspectroscopy (FTIRM), a technique by which microscopic analysis is performed within the infrared (IR) region of the spectrum. IR microspectroscopy has been possible ever since the introduction of interferometers using Fourier transformation some 30 years ago increased the sensitivity of IR spectroscopy by orders of magnitude. The spatial resolution of these instruments was limited to approximately 40 μm, however, because the aperture of the microscope masked the IR beam and essentially rejected a large proportion of the IR radiation. Additionally, the time involved in gathering spectra over a large area was prohibitive. With the introduction in the late 1990s of focal plane array detectors, consisting of large numbers of individual small detectors, both of these limitations were overcome and multiple IR spectra over large areas can now be taken close to the diffraction limit (10 μm at 1000 cm^-1^) [16]. With the instrument used in our studies, 4096 spectra of a sample area 34 μm^2 ^can be recorded simultaneously within seconds. Samples need to be thin (<10 μm) to allow the IR beam to penetrate the whole section. Here we have used the technique of absorption/reflection by mounting thin sections of tissue on slides coated with a thin layer of silver and tin oxide that reflects IR light but transmits visible light. Accordingly, the reflected beam passes twice through the sample, producing an array of IR spectra, and the visible light transparency allows correlation of each IR spectrum with a particular small area on the sample. At IR wavelengths, the spectra obtained are derived from vibrations within particular chemical bonds and provide information on the chemical composition of the tissue without need for specialized histochemical staining. According to the method of analysis used, images can be derived that represent the spectrum at a particular small region of the tissue, or chemical maps that represent the relative concentration of a particular analyte in different areas of the tissue. FTIRM is applicable to both paraffin-embedded tissue and cryosections, and thus can be combined with standard histological techniques. FTIRM has been previously applied to studies of cartilage and the spectra of collagens and proteoglycans are well defined [17-21].

## Materials and methods

### Monoclonal antibodies

CIIC1 [22] and M2139 [23] are arthritogenic mAbs that bind to well defined conformational epitopes on the CB11 and CB10 fragments of CII, respectively [8,10], and CIIF4 [22] is a nonarthritogenic mAb that binds to a conformational epitope on the CB9 fragment [8]. CIIC1 and M2139 either individually, or in combination, induce cartilage destruction after passive transfer [4]. GAD6 was a control mAb that binds to an irrelevant antigen glutamic acid decarboxylase [24]. CIIC1, CIIF4 and GAD6 are IgG2a, and M2139 is IgG2b. The mAbs CIIC1, M2139 and CIIF4 were derived from hybridomas derived from CII immunized mice and GAD6 was obtained from the Developmental Studies Hybridoma Bank maintained by the University of Iowa (Department of Biological Science, Iowa City, IA, USA). Hybridomas were cultured in miniPERM bioreactors (Heraeus Instruments, Hanau, Germany) in DMEM containing 10% (v/v) FCS (Trace Biosciences, Noble Park, Australia), 50 IU/ml penicillin and 50 mg/ml streptomycin as described previously [12]. The mAb quality was assessed using SDS-PAGE with 10% gels under reducing conditions, and the concentration of the mAbs was determined by densitometry against a sample of known concentration.

### F(ab)_2 _preparation

F(ab)_2 _was prepared from CIIC1 dialyzed against 0.2 M acetate buffer, pH 3.5, and digested with porcine pepsin at 37°C for 12 h. The digestion was terminated by dialyzing against PBS, pH 7.4, overnight, and the digest was passed through a protein A column to remove undigested mAb or Fc. The quality of the digestion and the concentration of F(ab)_2 _was determined by SDS-PAGE.

### Cultured bovine cartilage explants

Cartilage shavings from adult bovine metacarpal phalangeal joints were sliced into approximately 1 × 5 mm pieces; 50 mg of cartilage was used for each sample. The pieces were cultured at 37°C in the presence of 5% CO_2 _in 2 ml of DMEM containing 20% (v/v) heat-inactivated FCS containing 25 μg/ml ascorbic acid. The medium was changed every two days and fresh ascorbic acid and mAb were added at each change. Cartilage explants were cultured with mAbs (50 μg/ml) or medium alone for periods up to 21 days. To determine whether the effects were the result of Fc binding of the mAbs to chondrocytes, the explants were cultured with 100 μg/ml of F(ab)_2 _from CIIC1, an equivalent amount of intact CIIC1, or medium alone for 7 or 14 days.

### Immunofluorescence to detect antibody penetration

After 14 days in culture, cartilage explants were collected and snap frozen in OCT compound (Tissue-Tek, Sakura Finetechnical Co. Ltd, Tokyo, Japan) using dry ice and isopentane. Serial cryosections (5 μm) were stained with 0.1% toluidine blue in 30% ethanol, which stains the nuclei of the chondrocytes and the proteoglycans within the matrix to examine morphology, or treated by immunofluorescence to detect antibody penetration. For immunofluorescence, the sections were treated with 50 μl of type III hyaluronidase (Sigma, St. Louis, MO, USA) at 20 mg/ml in PBS for 30 minutes at room temperature, washed with PBS and incubated with sheep anti-mouse globulin conjugated with fluorescein isothiocyanate (FITC) (Silenus, Hawthorn, Australia) diluted 1:150 in carbonate buffer pH 8.6 containing 1% w/v BSA for 30 minutes at room temperature. To detect penetration of F(ab)_2_, the sections were incubated with a goat antibody to mouse F(ab)_2 _(ICN Biomedicals Inc., Aurora, OH, USA) diluted 1:2000 in PBS with 1% w/v BSA for 1 h, followed by incubation with rabbit anti-goat Ig, conjugated with Alexa 488 (Molecular Probes, Eugene, OR, USA) diluted 1:400 in carbonate buffer, pH 8.6, with 1% w/v BSA. The slides were then mounted with 90% v/v glycerol in PBS and observed microscopically.

### Histomorphometry

On selected days, cartilage explants were fixed in 4% paraformaldehyde, embedded in paraffin, and 5 μm sections were cut and stained with either toluidine blue or haemotoxylin and eosin. Histomorphometry was performed on MCID software (M4 3.0 Rev 1.1; Imaging Research Inc., St Catherines, Ontario, Canada). Images were captured at 200 × magnification from three to five separate pieces of tissue for each culture. At each timepoint, the mean loss of toluidine blue stain, mean chondron size, number of cells per mm^2 ^and the percentage of empty chondrons was determined. To determine the loss of toluidine blue staining from the section, the auto-select tool was used to designate and create a line at the point that the loss of staining ended; using the two-point straight-line measurement tool, the distance of loss was measured from the edge of the tissue, excluding any superficial layering, through to the line created by the auto-select tool. The measurement was performed six times on each image captured. MCID software was likewise used to measure the penetration of mAbs in the frozen sections. For chondron size, individual chondrons were manually outlined using the MCID software, which then calculated chondron area. An average of 24 chondrons (range ± 13) that contained cells (usually only one cell per chondron) were counted from each image, and empty chondrons were counted separately to calculate the percentage of empty chondrons. The number of cells per mm^2 ^was calculated by counting the number of cells within an area measured by the MCID software.

### Preparation of purified type II collagen and crude extract of proteoglycan for analysis by FTIRM

Bovine cartilage was treated with 4 M guanidine-HCl (Sigma). The resultant crude proteoglycan mixture, which contained predominantly aggrecan, was dialysed extensively against distilled water to remove guanidine-HCl and freeze dried. CII was prepared from the extracted cartilage by pepsin digestion and differential salt precipitation as previously described [7]. Ultra pure high molecular weight hyaluronan was provided by Garry Brownlee (Department of Biochemistry and Molecular Biology, Monash University, Clayton, Victoria, Australia).

### Measurement of changes in the composition of the matrix by Fourier transform infrared microspectroscopy

For the present study, 5 μm sections of paraffin embedded tissue taken at day 14 were placed onto MirrIR low-e microscope slides (Kevley Technologies, Chesterland, OH, USA), and adjacent sections were collected for staining with toluidine blue. Sections were dewaxed and allowed to air dry. To examine the spectra of CII, a crude proteoglycan extract and hyaluronan, 20 μl of each component was allowed to dry in air on a MirrIR microscope slide. FTIRM images were recorded with a 'Stingray' Digilab FTS 7000 series spectrometer (Digilab, Varian, Mulgrave, Victoria, Australia) coupled to a UMA 600 microscope equipped with a 64 × 64 focal plane array detector.

For each spectrum, 16 scans were co-added at a resolution of 6 cm^-1^. The spectra were preprocessed using purpose-built software compiled using Matlab (The Mathworks Inc., Natick, MA, USA) [25]. This processing entailed a linear base line correction and vector normalization. This data matrix was then exported into Cytospec Software for Infrared Spectral Imaging (Cytospec, Inc, , Berlin, Germany) and a 'quality test' was performed to remove spectra with poor signal-to-noise ratios and spectra containing obvious artifacts. Chemical maps were generated from the integrated intensities of specific functional groups identified in the spectra. Using the same software, 10 spectra from the antibody-exposed exterior of the explant, and from the interior of the explant, were extracted from the chemical maps. The mean spectrum for each was calculated to assess the effects of antibody penetration.

In the present study, we examined peaks characteristic of collagen and proteoglycans. An FTIRM spectrum of proteoglycans demonstrates peaks within the region of 1175-960 cm^-1 ^derived from carbohydrate moieties, and at 1241 cm^-1 ^derived from sulphate of the sulphated glycosaminoglycan side-chains [17,18]. The collagen spectrum shows a characteristic triplet of peaks at 1203, 1234 and 1280 cm^-1 ^but this region includes the peak at 1240–1245 cm^-1 ^characteristic of sulphates [17,18]. Accordingly, we examined the amide 1 peak that represents total protein, as a measure of the collagen content; the amide 1 peak for native collagen occurs at about 1666 cm^-1^, with a shift to a lower wave number (cm^-1^) on denaturation [21] or after collagenase treatment [26]. In the present study, these spectral shifts were confirmed using purified pepsin-digested CII prepared from bovine nasal cartilage [7], before and after heat denaturation at 50°C, and using explanted bovine cartilage treated with collagenase for 20 minutes before fixation and processing as described above.

### Statistical analysis

Statistical analyses were performed using Statistica for Windows, Version 4.5 (Statsoft Inc., Tulsa, OK, USA). ANOVA was performed to determine whether there were significant differences between groups, and Student's t-test, or the non-parametric Mann Whitney U-test, were used to compare individual differences. P < 0.05 was considered significant.

## Results

### Immunofluorescence to detect antibody penetration

**Figure 1:**
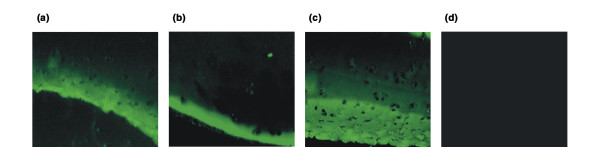
Immunofluorescence showing the penetration of the three anti-CII antibodies: **(a) **CIIF4, **(b) **CIIC1 and (c) M2139. The area of colour indicates antibody binding. **(d) **The control mAb (GAD6) shows no binding to the cartilage.

Each of the mAbs to CII, whether arthritogenic (CIIC1 and M2139) or not (CIIF4), penetrated the extracellular matrix during culture and remained bound to the tissue, as demonstrated by the areas of fluorescence around the edge of the explant, in contrast to the control mAb GAD6 (Fig. 1a–d). The distance (mean ± SD) of penetration at the surface of the cartilage was 48 ± 8 μm for CII-F4, 33 ± 8 μm for CIIC1 and 86 ± 8 μm for M2.139. The F(ab)_2 _of CIIC1 completely penetrated the tissue.

### Morphology of cartilage explants

**Figure 2:**
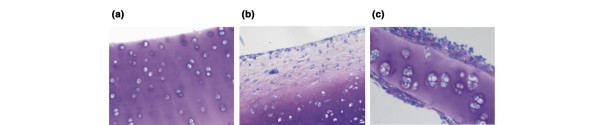
A toluidine blue stained sections of cartilage. **(a) **Cartilage cultured for 7 days shows an evenly stained matrix with typical rounded chondrocytes. Sections of cartilage incubated for **(b) **7 days and **(c) **14 days with M2139 show abnormal matrix morphology with the loss of toluidine blue, the development of a cellular layer at the surface and the development of hypertrophic chondrocytes.

As seen by light microscopy and toluidine blue staining, explants cultured in medium alone, or in the presence of either GAD6 or CIIF4, remained healthy even up to 21 days in culture (Fig. 2a), and stained strongly with toluidine blue. In contrast, the two arthritogenic mAbs, CIIC1 and M2139, caused profound changes in the explant structure, progressively over time. Both mAbs, and particularly M2139, had effects on the matrix. These included loss of toluidine blue staining from the surface of the tissue and, after 14 days in culture, development of a layer of cells on the surface of the explant (Fig. 2b, c). Chondrocytes developed changes resembling hypertrophy and there was a measurable increase in the proportion of empty chondrons. Notably, the non-arthritogenic mAb CIIF4 induced none of these changes.

**Figure 3:**
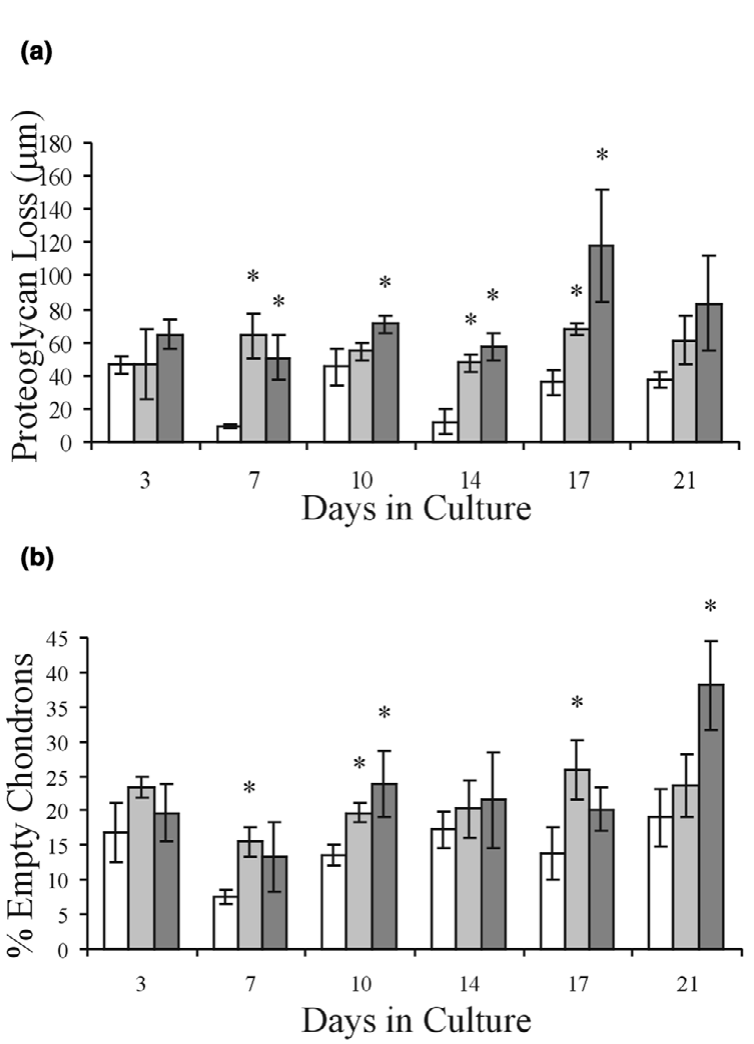
Differences in the loss of proteoglycan and chondrocyte between cultures incubated with different mABs. **(a) **Loss of toluidine blue staining between cultures incubated with CIIF4 (white) and cultures incubated with CIIC1 (light grey) and M2139 (dark grey) over the course of 21 days. **(b) **The number of empty chondrons expressed as a percentage of the total number of chondrons, indicating the loss of chondrocyte from the extracellular matrix. The columns represent the mean of each measurement and error bars indicate 1 standard deviation. The asterix represents p < 0.05.

To quantify changes in the explant structure, the loss of proteoglycans and percentages of empty chondrons were analyzed using culture samples collected at days 3, 7, 10, 14, 17 and 21. There were no significant differences by ANOVA in any of the measurements made between explants cultured individually with GAD6, CIIF4 or no antibody. For explants cultured with CIIC1, and particularly M2139, however, there was a significant increase in the loss of toluidine blue staining from the surface of the tissue over the period of culture that was not seen in the control groups. The controls, exemplified by CIIF4, a loss of staining similar to that for CIIC1 at day 4, but thereafter there was clear evidence of recovery (Fig. 3a). CIIC1, and particularly M2139, exhibited an increase in percentage of empty chondrons with increasing time in culture (Fig. 3b). There were no significant differences between either of the arthritogenic mAbs, CIIF4 or other controls in the number of cells per mm^2^, or in the size of the cells.

### Effect of F(ab)_2 _from CIIC1 on cartilage explants

To determine whether the changes observed were the result of direct antibody binding, or whether they resulted from binding of antibody complexes with Fc receptors on the surface of chondrocytes, the effects of the F(ab)_2 _fragment of CIIC1 were compared with those of intact CIIC1 after 7 or 14 days in culture. As seen by immunofluorescence, the F(ab)_2 _was able to penetrate a greater distance into the tissue than intact CIIC1 (data not shown), and the F(ab)_2 _caused greater disruption of architecture. Also there was greater loss of toluidine blue staining than for CIIC1.

### Fourier transform infrared spectra of cartilage components

**Figure 4:**
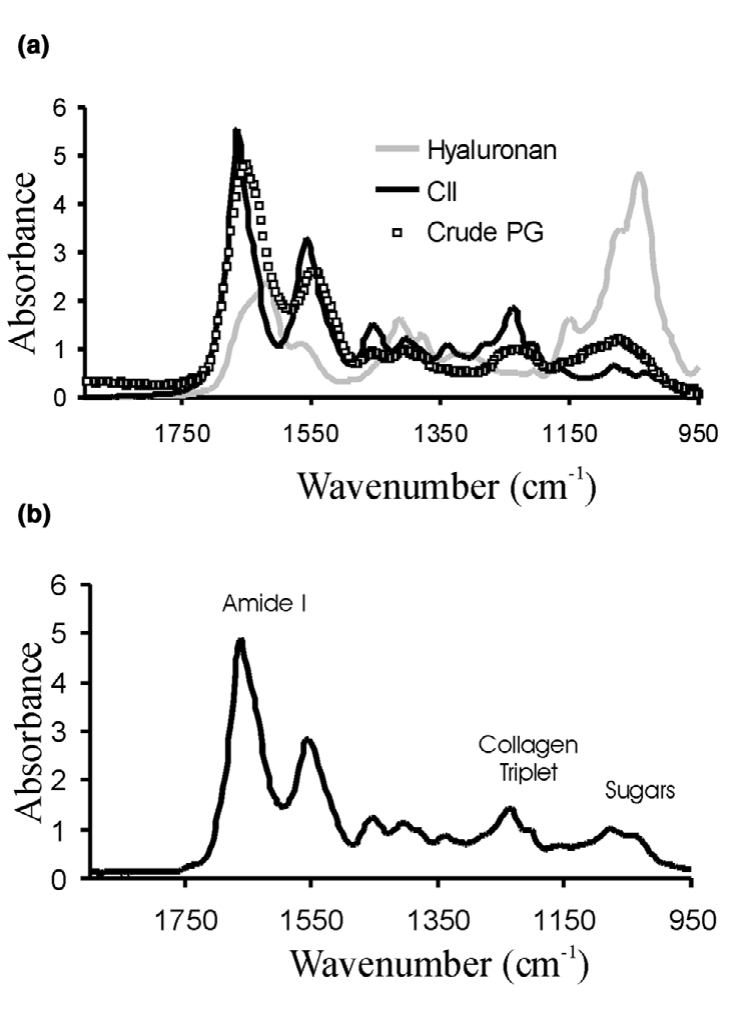
FTIRM spectra of the major cartilage components CII, proteoglycan and hyaluronan. **(a) **Typical spectra for CII, crude proteoglycan extract and hyaluronan. **(b) **An artificial spectrum that resembles normal articular cartilage generated by combining appropriate proportional amounts of the spectra of CII (55%), crude proteoglycan extract (40%) and hyaluronan (5%). The amide 1 peak from 1600–1700 cm^-1 ^represents the total protein content, the triplet of peaks from 1200–1300 cm^-1 ^are characteristic of the spectrum of collagen, and the peaks in the region 960–1175 cm^-1 ^result from sugars in the proteoglycans and hyaluronan.

The IR spectrum of a pure chemical is derived from vibrations within particular chemical bonds, and thus can provide a unique fingerprint for that chemical. In the case of complex biological systems, the spectrum derived is a composite of the individual spectra of the components of that tissue, and analysis of chemical changes depends on knowledge of the spectra of individual components. To validate the use of FTIRM in the present study, the spectra of the major cartilage components, CII, proteoglycan and hyaluronan were examined (Fig. 4a); each component had its own unique spectrum, establishing the ability of FTIRM to distinguish between these components. A combination of the spectra according to proportions that would represent those in articular cartilage, 55% collagen, 40% proteoglycan and 5% hyaluronan, generated a composite spectrum that resembled that of normal articular cartilage (Fig. 4b).

### Chemical changes in cartilage matrix detected by Fourier transform infrared microspectroscopy

**Figure 5:**
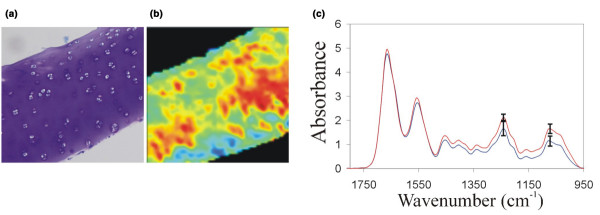
Distribution of proteoglycans in the cultured explants. **(a) **Toluidine blue stained sections cultured for 14 days with GAD6. **(b) **Chemical map derived using FTIRM showing the proteoglycan region (960–1175 cm^-1^). The chemical maps show the distribution and relative concentrations of proteoglycans; the least concentrated areas are shown as blue and the most concentrated areas that are shown as red. **(c) **The spectra shown are the mean of 10 measurements taken from either the central areas (red line) or near the surface of the tissue (blue line). The error bars represent 1 standard deviation at those points in the spectra. The amide 1 region, which represents the total protein content of the tissue, is from 1600–1700 cm^-1^.

The loss of proteoglycans observed from toluidine blue stained sections was confirmed by FTIRM. Information on proteoglycan distribution in the explants was generated by creating a chemical map made by integrating the area under the peaks in the 1175-960 cm^-1 ^region. Comparisons were made between serial sections of cartilage cultured in the presence of the control GAD6 (Fig. 5), stained with toluidine blue to show the distribution of proteoglycans (Fig. 5a), or processed by FTIRM (Fig. 5b), in which the regions with the highest concentration of proteoglycans are shown as red, and the lowest concentrations are shown as blue. In the section processed by FTIRM, the distribution of proteoglycans across the section was relatively even, with minimal loss of proteoglycans from the surface of the explant. This was confirmed by comparing mean spectra from the surface and middle of the section (Fig. 5c), although there was a slight reduction in proteoglycans at the edge of the section as shown by a reduction in the peak absorbance from the sugars (at 1072 cm^-1^) and a reduction in a peak at 1241 cm^-1 ^that is representative of the sulphate in the chondroitin and keratan sulphates of proteoglycans (Table 1). The distribution of proteoglycans and the spectra obtained for GAD6 were characteristic of those obtained for cartilage cultured without antibody.

**Figure 6:**
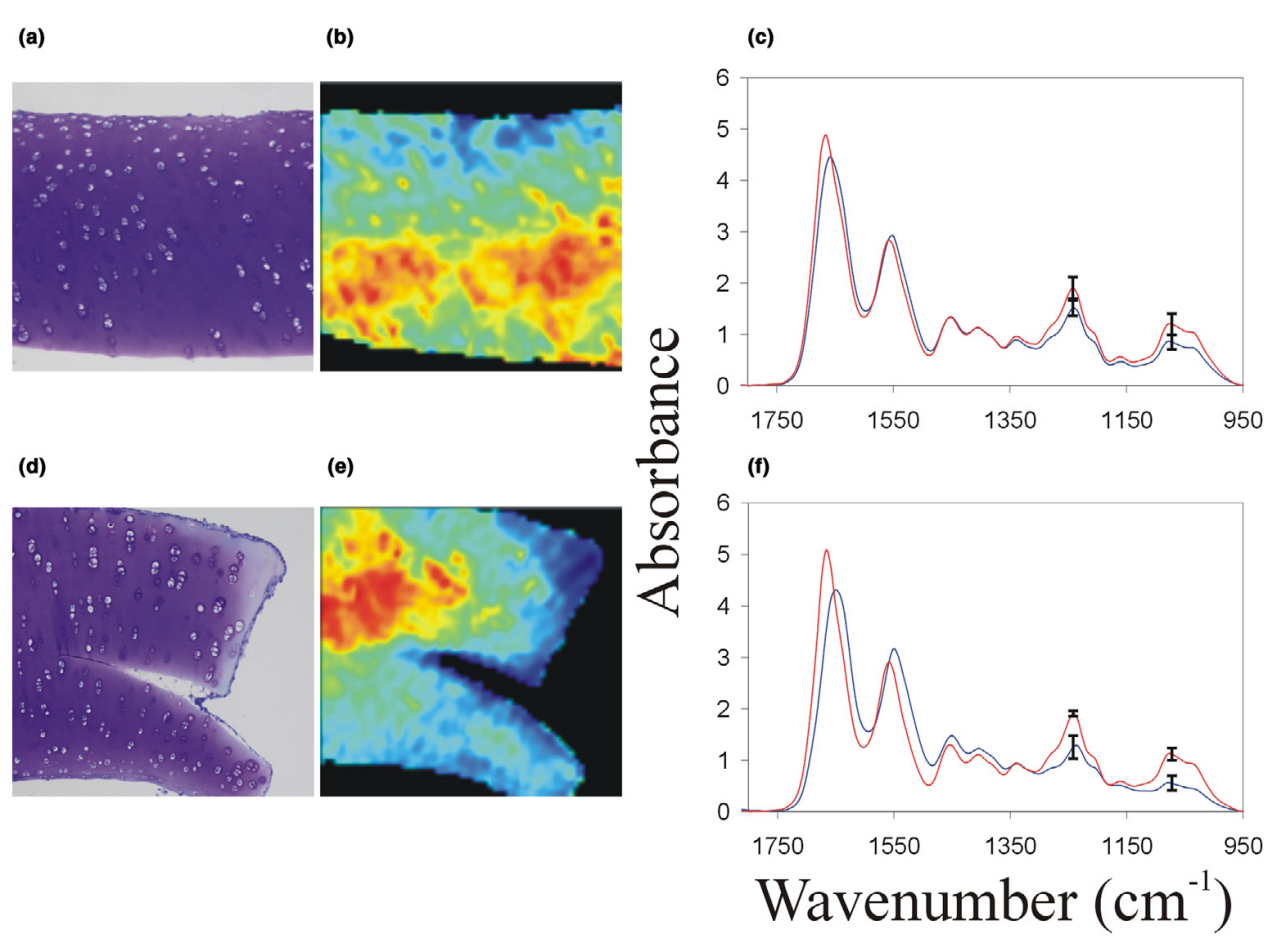
Distribution of proteoglycans in the explants cultured with CIIF4 or M2139. Toluidine blue stained sections cultured for 14 days with **(a) **CIIF4 or **(d) **M2139 are shown alongside **(b, e) **chemical maps showing proteoglycan distribution and **(c, f) **FTIRM spectra from the central areas (red line) and near the surface of the tissue (blue line). The error bars represent 1 standard deviation at those points in the spectra.

**Figure 7:**
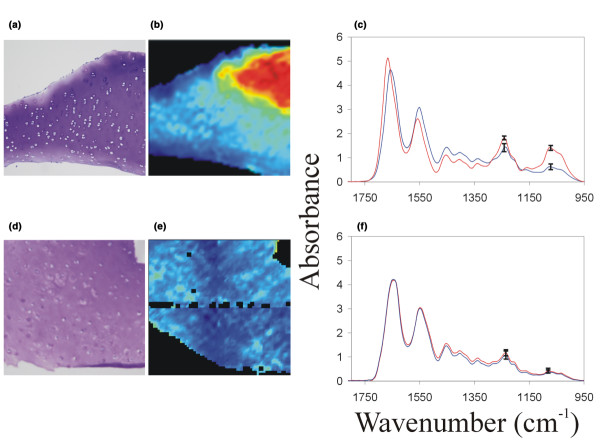
Distribution of proteoglycans in the explants cultured with CII-C1 or F(ab)_2 _from CII-C1. Toluidine blue stained sections cultured for 14 days with **(a) **CII-C1 or **(d) **F(ab)_2 _are shown alongside **(b, e) **chemical maps showing proteoglycan distribution and **(c, f) **FTIRM spectra from the central areas (red line) and near the surface of the tissue (blue line). Note that the proteoglycan levels are lower and the amide 1 peak has shifted across the whole of the F(ab)_2 _treated tissue. The error bars represent 1 standard deviation at those points in the spectra.

In contrast, there were marked differences in the distribution of proteoglycans across the tissue for explants cultured with each of the mAb to CII. The mean spectra taken at the surface of the section as well as those from the middle also differed (Figs 6a–f and 7a–c; Table 1). The concentration of proteoglycans from the middle of the tissue, beyond the penetration of the mAb, did not differ from the controls, as judged by the height of the peaks at 1175-960 cm^-1^, and peaks at 1203, 1234 and 1280 cm^-1^; therefore, spectra (n = 10) from the interior of the cartilage treated with the four mAbs were combined (Table 1). There was, however, a reduction in the concentration of proteoglycans at the surface of the tissue based on the decrease in the peak at 1072 cm^-1^, and a corresponding decrease in the sulphate peak at 1241 cm^-1 ^that was much greater for CIIC1 and M2139 than for CIIF4 (Table 1). In addition, at the surface of the tissue in each of the explants treated with the mAbs to CII, but not with GAD6, there was a decrease in absorbance and a spectral shift in amide 1, from a peak at 1666 cm^-1 ^to below 1660 cm^-1 ^(Table 1). These results are consistent with the spectral shifts in the amide 1 peak, obtained after heat denaturation of purified CII (from 1666 to 1652 cm^-1^) and with surface changes observed after treatment of cartilage with collagenase (from 1668 to 1653 cm^-1^)

The F(ab)_2 _treated cartilage showed a uniform and substantial loss of proteoglycan across the toluidine blue-stained tissue (Fig. 7d, e); this was confirmed by the mean spectra from the surface and from the middle of the section (Fig. 7f). There was almost complete loss of the proteoglycan peak between 1175-960 cm^-1^, a marked reduction in the sulphate peak at 1241 cm^-1^, and the peaks at 1203, 1234 and 1280 cm^-1^, and a striking decrease and spectral shift to 1644 cm^-1 ^in the amide 1 peak, indicative of denaturation and loss of CII from the matrix, across the whole tissue (Table 1).

## Discussion

Human RA and its animal model CIA are complex diseases in which both the immune response and subsequent inflammation are important determinants of cartilage destruction. The effector phase of CIA, evident two to three days after passive transfer of anti-CII to healthy mice has been studied extensively [3,4,9,10,27,28], and the development of collagen antibody-induced arthritis provides an informative *in vivo *model in which inflammatory processes can be examined in the absence of an inductive immune response [4]. Little is known, however, about any direct effects of antibody on the target cartilage tissue and the contribution of this to the ensuing disease process. Our study has investigated the effects of two such arthritogenic mAbs to CII, CIIC1 and M2139, on cultured bovine cartilage explants and compared the results with a non-arthritogenic mAb to CII, CIIF4, and to an irrelevant mAb, GAD6, in the absence of inflammatory mediators known to dominate the effector phase of CIA. Both arthritogenic and non-arthritogenic mouse mAbs to CII were shown by immunofluorescence to penetrate cartilage and bind strongly to the matrix, but only the former had adverse effects. Such binding caused loss and denaturation of collagen. Loss of proteoglycans was observed both by light microscopy as loss of toluidine blue staining of the matrix, and changes in the chemical map by FTIRM. Concomitantly, we observed the appearance of 'empty' chondrons in the cartilage, and the development of a superficial cell layer of morphologically non-descript cells within a matrix that reacted strongly to immunofluorescence at day 14 of culture. Such effects could explain the observation that not all antibodies to CII are arthritogenic, and pathogenicity may depend on the particular epitope(s) recognized [8,29].

FTIRM has emerged over the last 10 years as a most effective means of identifying and quantifying differences between defined areas or single points of histological specimens, and the present study illustrates its use for the examination of changes in the collagen content of the cartilage that would otherwise require complex quantitative biochemical analysis [12,30] or multiple immunohistochemical studies [31]. The shift in the amide 1 peak in the areas penetrated by antibody in explants cultured with both the arthritogenic mAbs (CIIC1 and M2139) and the non-arthritogenic mAb CIIF4 is consistent with changes that occur during denaturation of collagen [21] or during collagenase treatment [26], and that are taken to represent an unwinding of the triple helical conformation. Notably, in the same areas, a reduction in the levels of collagen was shown by a reduction in the collagen triplet between 1300-1200 cm^-1^. Finally, as seen by the reduction of peaks in the range 1175-960 cm^-1^, FTIRM confirmed the reduction of proteoglycans shown by toluidine blue staining that occurred around the surface of the cartilage explants exposed to CIIC1 and M2139, and the complete loss of proteoglycan in explants exposed to F(ab)_2 _of CIIC1. The changes seen *in vitro *are similar to changes that occur in cartilage *in vivo *after passive transfer of mAbs, although such changes *in vivo *are assumed to be due to the effects of degradative enzymes produced by the accompanying inflammation. It is of interest that loss of proteoglycan is an early marker of the cartilage disruption that occurs in both osteoarthritis [32] and RA [33], in which it is attributed to matrix metalloproteinases and aggrecanases (the ADAMTS or 'a disintegrin and metalloproteinase with thrombospondin motif' family of proteases) [31,32] that are released following disruption of the molecular interactions between matrix constituents. This loss of the proteoglycans, which provide 'cushioning' of the cartilage in the joint, in turn allows a greater susceptibility to damage from compressive forces and greater penetration of degradative molecules.

The changes in the matrix at the surface of the cartilage observed in the explants cultured with the arthritogenic mAbs CIIC1 and M2139 accompanied appearances of 'empty' chondrons in the cartilage. Although apoptosis is a common secondary effect of cartilage disruption, it is difficult to measure in cartilage. We therefore used loss of chondrocytes from the matrix as a measure of cell death, as has been done before [34]. Cultures with CIIC1, and particularly M2139, demonstrated increasing numbers of empty chondrons over time and, in many cases, the same sections showed chondrons containing several cells, which is suggestive of hyperplasia as a compensatory response to mAb-mediated cartilage damage. By day 14 of culture the surface of explants exposed to CIIC1 or M2139 had a superficial layer of morphologically non-descript cells within a scanty matrix. Presumably this cell layer, having lost the proteoglycans, was composed of collapsed cartilage. Strong staining by immunofluorescence, which demonstrated the presence of CII, provided further evidence that this layer had a cartilaginous origin. Its appearance was suggestive of the fibrous pannus characteristically described in rheumatoid arthritis, which has also been shown to contain CII, and is also possibly derived from chondrocytes [35,36].

The use of F(ab)_2 _demonstrated that the effects we observed with cultured explants is not Fc mediated. Evidence that chondrocytes express Fc receptors is limited, but non-specific Fc-mediated binding of immune complexes to chondrocytes has been reported to stimulate matrix metalloproteinase production and production of interleukin 1 by chondrocytes [37]. While Fc receptors have been shown to be important in CIA [38,39], particularly in inflammation induction and in the passive transfer of antibody-mediated disease [10], successful treatment of inflammation can still leave an ongoing problem of continuing joint destruction [40-42]. In the present study, the effect of the F(ab)_2 _was much greater than that of a corresponding molar concentration of intact CIIC1. This is because the smaller size of the F(ab)_2 _would allow it to penetrate more deeply into the tissue; indeed, in F(ab)_2_-treated sections, there was a correspondingly greater loss of proteoglycans and decreased collagen content as seen by FTIRM. Alternatively, if Fc-receptor binding were, in fact, a normal physiological method of removing immune complexes [43], mAb bound to collagen in the cultured tissue could persist, and the total amount of mAb could increase with each change of medium. If this is the case, then the greater effects caused by the F(ab)_2 _would truly represent the effect of having higher amounts of antibody.

We emphasize that all of the changes in this study have been observed *in vitro *without the confounding influences of inflammation, complement and other immunological mediators present in a CIA or RA- affected joint, and that the use of a F(ab)_2 _fragment of the arthritogenic mAb excludes the possibility of these effects being due to Fc binding. Presently, the assessment of cartilage damage in CIA relies on scoring joint damage, histological abnormalities and measuring release of cartilage breakdown products such as cartilage oligomeric matrix protein [44]. This could mask the damaging effect of antibody binding; denaturation of collagen in the matrix that leads to disruption in the organization of the matrix. Our results suggest that the effects of arthritogenic mABs on *de novo *synthesis of cartilage matrix that we have previously reported from studies on chondrocyte cultures [12] are paralleled by degradative effects on preexisting cartilage. These include not only the loss of matrix, but also loss of chondrocytes and denaturation of collagen fibrils and would contribute to direct and on-going cartilage loss that is independent of any injurious effect of inflammation. This is in accord with the likelihood that loss of cartilage in RA, seen radiologically as joint space narrowing, may be due to a different process than that responsible for development of erosions [45].

## Conclusion

This study has important connotations for our understanding of the pathogenesis of RA. Autoantibodies to collagen occur in RA [5-7], bind to cartilage and can be released from immune complexes within the cartilage by treatment with collagenase [46], and have specificity for epitopes that are arthritogenic in mice [8]. Both CIA and RA are complex polygenic diseases in which the gross pathology results from cell- and antibody-mediated inflammation. We have also demonstrated that arthritogenic mAbs to CII can contribute directly to cartilage destruction, which implies the involvement of non-inflammatory as well as inflammatory components in the disease process. It is even possible that injurious effects of antibody on articular cartilage may precede and even initiate subsequent inflammatory events that contribute to ultimate joint destruction, and provides a further rationale for the successful use of combination therapies [47].

## Abbreviations

BSA = bovine serum albumin; CIA = collagen induced arthritis; CII = type II collagen; DMEM = Dulbecco's modified Eagle's medium; FCS = fetal calf serum; FITC = fluorescein isothiocyanate; FTIRM = Fourier transform infrared microspectroscopy; IR = infrared; mAB = monoclonal antibody; PBS phosphate buffered saline; RA = rheumatoid arthritis.

## Competing interests

The authors declare that they have no competing interests.

## Authors' contributions

DEC carried out explant and hybridoma cultures, immunofluorescence, performed MCID and FTIRM analysis and drafted the manuscript. MT developed the explant culture system and performed the initial experiments. BBZ prepared F(ab)_2 _and tested its effects on cultures. BW and DMcN were responsible for the analysis and interpretation of the FTIRM results. KSN and RH provided the monoclonal antibodies used in the study, and have revised the manuscript critically for intellectual content based on experience with the *in vivo *animal model. MPVD provided expertise with chondrocyte and explant cultures, participated in the design of the study, and helped draft the manuscript. MJR conceived of the study, participated in its design and coordination, performed statistical analysis and helped draft the final manuscript. All authors read and approved the final manuscript.

## Figures and Tables

**Table 1:** Absorbance data from FTIRM used to examine levels of proteoglycan and collagen from different cultures

Sample (no. of spectra)	Proteoglycan absorbance		Amide 1 peak^c^	
	1072 cm^-1a^	1242 cm^-1b^	Absorbance	Location (cm^-1^)
Interior (40)^d^	1.34 ± 0.26	1.94 ± 0.17	5.01 ± 0.15	1666
GAD 6 edge (10)	1.14 ± 0.21	1.66 ± 0.20	4.76 ± 0.44	1666
CIIF4 edge (10)	0.85 ± 0.14	1.51 ± 0.15	4.46 ± 0.19	1659
M2139 edge (10)	0.55 ± 0.14	1.25 ± 0.23	4.31 ± 0.19	1651
CIIC1 edge (10)	0.49 ± 0.12	1.42 ± 0.17	4.63 ± 0.41	1659
M2139 + CIIC1 edge	0.37 ± 0.11	0.99 ± 0.19	4.44 ± 0.15	1639
M2139 + CIIC1 middle	0.50 ± 0.14	1.05 ± 0.15	4.48 ± 0.10	1643
CIIC1 F(ab)_2 _edge (10)	0.40 ± 0.07	1.03 ± 0.17	4.22 ± 0.14	1648
CIIC1 F(ab)_2 _interior (10)	0.43 ± 0.1	1.12 ± 0.12	4.18 ± 0.14	1644

^a^Absorbance from the proteoglycan peak at 1072 cm^-1 ^is representative of sugars. ^b^Absorbance from the proteoglycan peak at 1242 cm^-1 ^is representative of sulphated glycosaminoglycans. ^c^The amide 1 peak provides a measure of total protein, predominantly collagen. Note the change in the location of the amide 1 peak in the presence of mAbs to CII, consistent with the change from 1666–1668 cm^-1 ^to 1652–1653 cm^-1 ^observed after heat denaturation of the collagen helix, or collagenase treatment (see text). Results shown are mean ± SD. ^d^Ten spectra from the interior of the cartilage treated with the four antibodies were combined.

## References

[B1] Trentham DE, Townes AS, Kang AH (1977). Autoimmunity to type II collagen an experimental model of arthritis. J Exp Med.

[B2] Courtenay JS, Dallman MJ, Dayan AD, Martin A, Mosedale B (1980). Immunisation against heterologous type II collagen induces arthritis in mice. Nature.

[B3] Stuart JM, Cremer MA, Townes AS, Kang AH (1982). Type II collagen-induced arthritis in rats. Passive transfer with serum and evidence that IgG anticollagen antibodies can cause arthritis. J Exp Med.

[B4] Nandakumar KS, Svensson L, Holmdahl R (2003). Collagen type II-specific monoclonal antibody-induced arthritis in mice: description of the disease and the influence of age, sex, and genes. Am J Pathol.

[B5] Ronnelid J, Lysholm J, Engstrom-Laurent A, Klareskog L, Heyman B (1994). Local anti-type II collagen antibody production in rheumatoid arthritis synovial fluid. Evidence for an HLA-DR4-restricted IgG response. Arthritis Rheum.

[B6] Cook AD, Rowley MJ, Stockman A, Muirden KD, Mackay IR (1994). Specificity of antibodies to type II collagen in early rheumatoid arthritis. J Rheumatol.

[B7] Cook AD, Gray R, Ramshaw J, Mackay IR, Rowley MJ (2004). Antibodies against the CB10 fragment of type II collagen in rheumatoid arthritis. Arthritis Res Ther.

[B8] Burkhardt H, Koller T, Engstrom A, Nandakumar KS, Turnay J, Kraetsch HG, Kalden JR, Holmdahl R (2002). Epitope-specific recognition of type II collagen by rheumatoid arthritis antibodies is shared with recognition by antibodies that are arthritogenic in collagen-induced arthritis in the mouse. Arthritis Rheum.

[B9] Terato K, Hasty KA, Reife RA, Cremer MA, Kang AH, Stuart JM (1992). Induction of arthritis with monoclonal antibodies to collagen. J Immunol.

[B10] Nandakumar KS, Andren M, Martinsson P, Bajtner E, Hellstrom S, Holmdahl R, Kleinau S (2003). Induction of arthritis by single monoclonal IgG anti-collagen type II antibodies and enhancement of arthritis in mice lacking inhibitory FcgammaRIIB. Eur J Immunol.

[B11] Gray RE, Seng N, Mackay IR, Rowley MJ (2004). Measurement of antibodies to collagen II by inhibition of collagen fibril formation in vitro. J Immunol Methods.

[B12] Amirahmadi SF, Pho MH, Gray RE, Crombie DE, Whittingham SF, Zuasti BB, Van Damme M-P, Rowley MJ (2004). An arthritogenic monoclonal antibody to type II collagen, CII-C1, impairs cartilage formation by cultured chondrocytes. Immunol Cell Biol.

[B13] Amirahmadi SF, Whittingham SF, Nandakumar KS, Holmdahl R, Mackay IR, Van Damme MP, Rowley MJ (2005). Arthritogenic anti-type II collagen antibodies are pathogenic for cartilage-derived chondrocytes independent of inflammatory cells. Arthritis Rheum.

[B14] Verzijl N, DeGroot J, Thorpe SR, Bank RA, Shaw JN, Lyons TJ, Bijlsma JW, Lafeber FP, Baynes JW, TeKoppele JM (2000). Effect of collagen turnover on the accumulation of advanced glycation end products. J Biol Chem.

[B15] Holmdahl R, Mo JA, Jonsson R, Karlstrom K, Scheynius A (1991). Multiple epitopes on cartilage type II collagen are accessible for antibody binding in vivo. Autoimmunity.

[B16] Bhargava R, Levin IW (2001). Fourier transform infrared imaging: a new spectroscopic tool for microscopic analyses of biological tissue. Trends Appl Spectr.

[B17] Camacho NP, West P, Torzilli PA, Mendelsohn R (2001). FTIR microscopic imaging of collagen and proteoglycan in bovine cartilage. Biopolymers.

[B18] Potter K, Kidder LH, Levin IW, Lewis EN, Spencer RG (2001). Imaging of collagen and proteoglycan in cartilage sections using Fourier transform infrared spectral imaging. Arthritis Rheum.

[B19] George A, Veis A (1991). FTIRS in H2O demonstrates that collagen monomers undergo a conformational transition prior to thermal self-assembly in vitro. Biochemistry.

[B20] Lazarev YA, Grishkovsky BA, Khromova TB (1985). Amide I band of IR spectrum and structure of collagen and related polypeptides. Biopolymers.

[B21] Payne KJ, Veis A (1988). Fourier transform IR spectroscopy of collagen and gelatin solutions: deconvolution of the amide I band for conformational studies. Biopolymers.

[B22] Holmdahl R, Rubin K, Klareskog L, Larsson E, Wigzell H (1986). Characterization of the antibody response in mice with type II collagen-induced arthritis, using monoclonal anti-type II collagen antibodies. Arthritis Rheum.

[B23] Mo JA, Holmdahl R (1996). The B cell response to autologous type II collagen: biased V gene repertoire with V gene sharing and epitope shift. J Immunol.

[B24] Chang YC, Gottlieb DI (1988). Characterization of the proteins purified with monoclonal antibodies to glutamic acid decarboxylase. J Neurosci.

[B25] Romeo MJ, Wood BR, Quinn MA, McNaughton D (2003). Removal of blood components from cervical smears: implications for cancer diagnosis using FTIR spectroscopy. Biopolymers.

[B26] Federman S, Miller LM, Sagi I (2002). Following matrix metalloproteinases activity near the cell boundary by infrared micro-spectroscopy. Matrix Biol.

[B27] Terato K, Harper DS, Griffiths MM, Hasty DL, Ye XJ, Cremer MA, Seyer JM (1995). Collagen-induced arthritis in mice: synergistic effect of E. coli lipopolysaccharide bypasses epitope specificity in the induction of arthritis with monoclonal antibodies to type II collagen. Autoimmunity.

[B28] Watson WC, Brown PS, Pitcock JA, Townes AS (1987). Passive transfer studies with type II collagen antibody in B10.D2/old and new line and C57Bl/6 normal and beige (Chediak-Higashi) strains: evidence of important roles for C5 and multiple inflammatory cell types in the development of erosive arthritis. Arthritis Rheum.

[B29] Myers LK, Rosloniec EF, Cremer MA, Kang AH (1997). Collagen-induced arthritis, an animal model of autoimmunity. Life Sci.

[B30] Beesley JE, Jessup E, Pettipher R, Henderson B (1992). Microbiochemical analysis of changes in proteoglycan and collagen in joint tissues during the development of antigen-induced arthritis in the rabbit. Matrix.

[B31] Fosang AJ, Stanton H, Little CB, Atley LM (2003). Neoepitopes as biomarkers of cartilage catabolism. Inflamm Res.

[B32] Lin PM, Chen CT, Torzilli PA (2004). Increased stromelysin-1 (MMP-3), proteoglycan degradation (3B3- and 7D4) and collagen damage in cyclically load-injured articular cartilage. Osteoarthritis Cartilage.

[B33] Mitchell NS, Shepard N (1978). Changes in proteoglycan and collagen in cartilage in rheumatoid arthritis. J Bone Joint Surg Am.

[B34] Aigner T, Kim HA (2002). Apoptosis and cellular vitality: issues in osteoarthritic cartilage degeneration. Arthritis Rheum.

[B35] Allard SA, Maini RN, Muirden KD (1988). Cells and matrix expressing cartilage components in fibroblastic tissue in rheumatoid pannus. Scand J Rheumatol Suppl.

[B36] Allard SA, Muirden KD, Camplejohn KL, Maini RN (1987). Chondrocyte-derived cells and matrix at the rheumatoid cartilage-pannus junction identified with monoclonal antibodies. Rheumatol Int.

[B37] Saura R, Uno K, Satsuma S, Kurz EU, Scudamore RA, Cooke TD (1993). Mechanisms of cartilage degradation in inflammatory arthritis: interaction between chondrocytes and immunoglobulin G. J Rheumatol.

[B38] Diaz de Stahl T, Andren M, Martinsson P, Verbeek JS, Kleinau S (2002). Expression of FcgammaRIII is required for development of collagen-induced arthritis. Eur J Immunol.

[B39] Hogarth PM (2002). Fc receptors are major mediators of antibody based inflammation in autoimmunity. Curr Opin Immunol.

[B40] Joosten LA, Helsen MM, Saxne T, van De Loo FA, Heinegard D, van Den Berg WB (1999). IL-1 alpha beta blockade prevents cartilage and bone destruction in murine type II collagen-induced arthritis, whereas TNF-alpha blockade only ameliorates joint inflammation. J Immunol.

[B41] Mulherin D, Fitzgerald O, Bresnihan B (1996). Clinical improvement and radiological deterioration in rheumatoid arthritis: evidence that the pathogenesis of synovial inflammation and articular erosion may differ. Br J Rheumatol.

[B42] Landewe R, Geusens P, Boers M, van der Heijde D, Lems W, te Koppele J, van der Linden S, Garnero P (2004). Markers for type II collagen breakdown predict the effect of disease-modifying treatment on long-term radiographic progression in patients with rheumatoid arthritis. Arthritis Rheum.

[B43] van Lent P, Nabbe KC, Boross P, Blom AB, Roth J, Holthuysen A, Sloetjes A, Verbeek S, van den Berg W (2003). The inhibitory receptor FcgammaRII reduces joint inflammation and destruction in experimental immune complex-mediated arthritides not only by inhibition of FcgammaRI/III but also by efficient clearance and endocytosis of immune complexes. Am J Pathol.

[B44] Vingsbo-Lundberg C, Nordquist N, Olofsson P, Sundvall M, Saxne T, Pettersson U, Holmdahl R (1998). Genetic control of arthritis onset, severity and chronicity in a model for rheumatoid arthritis in rats. Nat Genet.

[B45] Kirwan J, Byron M, Watt I (2001). The relationship between soft tissue swelling, joint space narrowing and erosive damage in hand X-rays of patients with rheumatoid arthritis. Rheumatology (Oxford).

[B46] Jasin HE (1985). Autoantibody specificities of immune complexes sequestered in articular cartilage of patients with rheumatoid arthritis and osteoarthritis. Arthritis Rheum.

[B47] Klareskog L, van der Heijde D, de Jager JP, Gough A, Kalden J, Malaise M, Martin Mola E, Pavelka K, Sany J, Settas L (2004). Therapeutic effect of the combination of etanercept and methotrexate compared with each treatment alone in patients with rheumatoid arthritis: double-blind randomised controlled trial. Lancet.

